# Plasticity in extended phenotype increases offspring defence despite individual variation in web structure and behaviour

**DOI:** 10.1016/j.anbehav.2018.01.022

**Published:** 2018-03-20

**Authors:** Nicholas DiRienzo, Hitoshi Aonuma

**Affiliations:** a Department of Ecology and Evolutionary Biology, University of Arizona, Tucson, AZ, U.S.A; b Research Institute for Electronic Science, Hokkaido University, Sapporo, Hokkaido, Japan; c CREST, Japan Science and Technology Agency, Tokyo, Japan

**Keywords:** animal architecture, animal personality, extended phenotype, offspring defence, parental investment

## Abstract

Many animals actively defend their offspring using a range of behaviours from calling and mobbing in birds, to physical grappling in crustaceans, and the expression of these behaviours positively scale with offspring value. While this role of behaviour in defence is well studied, very little is known about how other traits, specifically the structure of architectural constructions such as webs and nests, contribute to offspring defence. Additionally, although some tax a show consistent individual differences in offspring defence behaviour, it is completely unknown whether individuals also differ in defensive structures. We addressed these questions in the redback spider, *Latrodectus hasselti*, by measuring how a female laying an eggcase influences female behaviour and web structure, and whether those traits scale with relative reproductive investment. Our results show that females modified web structure in response to an eggcase, but only the protective elements of web structure positively scaled with the relative value of that eggcase. Finally, despite the significant correlations, fixed effects (e.g. eggcase possession/value) in the models explained only 5–23% of the variation in behaviour and web structure, while the random effect of individual identity explained 46–65% of the variation. This variation drove moderate to high repeatability estimates across all traits, suggesting that some individuals consistently invest relatively more in defence, while some invest less. These results highlight that extended phenotypic traits may be a critical component of offspring defence in some taxa. Furthermore, individual variation in these traits suggest that different reproductive strategies may exist, whereby some individuals invest more in reproduction at a cost to safety/foraging and vice versa.

The juvenile stage of virtually all animals is frequently the most vulnerable to predation. Behavioural defences are one method by which many species with some level of parental care have evolved to protect offspring from predation (Andersson, Wiklund, & Rundgren, 1980; Montgomerie & Weatherhead, 1988). Furthermore, it is hypothesized that organisms should scale their level of defence to the relative value of their offspring, investing more if there are larger brood numbers or as the brood ages (Andersson et al., 1980; Patterson, Petrinovich, & James, 1980). Although both of these points have been the focus of extensive research, virtually all the studies have focused on active behavioural defence mechanisms (e.g. aggression), while overlooking the role architectural constructions may play in defence. Additionally, we now know that almost all taxa show some level of consistent individual differences in behaviour (e.g. arachnids: Pruitt, DiRienzo, Kralj-Fišer, Johnson, & Sih, 2011; insects: Niemelä, DiRienzo, & Hedrick, 2012; birds: Schuett, Godin, & Dall, 2011; mammals: Guenther, Finkemeier, & Trillmich, 2014; fish: Bell & Sih, 2007), thus prompting the question of whether individuals also vary in how they defend their offspring. Individual variation in structural defences may provide a new level at which fitness trade-offs may occur, while also explaining how trait variation is maintained in populations. Despite these potentially important interactions, to our knowledge no study has investigated the interplay between behaviour, extended phenotypes and individual variation.

Offspring, especially those who are immobile, are vulnerable to a wide variety of biotic threats. The most common of these threats comes from predators, to which parents often defend against using a variety of aggressive behaviours, from mobbing and alarm calling in avian species (Hollander, Van Overveld, Tokka, & Matthysen, 2008; Redondo & Carranza, 1989; Wiklund, 1990), to biting and lunging in fish (Itzkowitz, 1985; Ridgway, 1988), to striking and grabbing with chelae in crayfish (Figler, Blank, & Peeke, 2001; Martin & Moore, 2010). Similar behaviours are used to counter other biotic threats such infanticidal females (Wolff & Peterson, 1998) and brood parasites (Trnka, Požgayová, Samaš, & Honza, 2013). Defending against these threats yields obvious fitness benefits in the form of offspring survival, yet comes at a potential cost of injury or death to parents. The reproductive value hypothesis predicts that parents should scale their defensive efforts relative to the value of the offspring (Patterson et al., 1980). Indeed, evidence in support of this hypothesis has been found in a number of species (Greig-Smith, 1980; Redondo & Carranza, 1989; Ridgway, 1988; Wiklund, 1990). For example, in merlins, *Falco columbarius*, female parents increase attack intensity as brood size increases, while also increasing defence for first broods, which have higher survival probability, compared to replacement broods, which have lower survival probability (Wiklund, 1990). Thus, the increased risk of injury associated with vigorous defence is offset by the predicted increase in reproductive fitness.

Although these behavioural mechanism yield fitness benefits in the form of increased offspring survival, there are other forms of defence that may provide similar protection and potentially interact with behavioural mechanisms. Animal constructions, which are extended phenotypic traits that include structures such as spider webs, ant and bee nests and beaver dams, mediate a number of fitness-related processes (e.g. foraging, mating, defence; Doucet & Montgomerie, 2003; Pinter-Wollman, 2015; Smith, Ostwald, & Seeley, 2015), including, potentially, offspring defence. To date, much of the work has focused on how factors such as nest site selection, density and conspicuousness of the construction may aid in defence (for review see Mainwaring, Hartley, Lambrechts, & Deeming, 2014), while overlooking how the architecture itself may do so. Certain features may be more effective at defence than others. For example, male sand gobys, *Pomatoschistus minutus*, were shown to reduce the size of their nest opening, which is used to aerate their brood, when predators are present, suggesting a defensive function of altering opening size (Lissåker & Kvarnemo, 2006). Such variation in architecture in response to investing in reproduction may be common, while also interacting with behaviour. Furthermore, given that these structures are costly to produce, both energetically and in terms of consequences for the offspring (Ford, 1977; Jakob, 1991; Lissåker & Kvarnemo, 2006), they also may be subject to the reproductive value hypotheses, whereby individuals scale the protective capability of their structure in accordance with the value of their offspring (Patterson et al., 1980).

While animals should increase their defensive efforts, both behaviourally and structurally, when they have offspring, as well as scale those defences with offspring value, extensive research has shown that animals are often limited in their plasticity. Such limits result in individuals displaying consistently different responses to the same context or situation (Sih, Bell, & Johnson, 2004). Some spiders are consistently more aggressive towards prey (DiRienzo & Montiglio, 2016a), mosquito fish and social spiders vary in how social they are (Cote, Fogarty, & Sih, 2012; Pruitt, Riechert, & Jones, 2008), and field crickets vary in their boldness (DiRienzo, Niemelä, Skog, Vainikka, & Kortet, 2015). Such individual differences may also occur in the context of nest defence, whereby some individuals invest more in protecting their offspring than others (Burtka & Grindstaff, 2013). Recently, a number of studies on avian species have demonstrated such individual differences in behavioural nest defences, measured by repeatability, whereby individual females are consistent across breeding seasons in how intensely they defend their nests (Burtka & Grindstaff, 2013; Hollander et al., 2008; Trnka et al., 2013). This raises the question of how those individuals who consistently invest less in behavioural defence compensate for the potential fitness losses. One hypothetical explanation for the maintenance of nonaggressive females in this population is that they build different structures that better protect their offspring, thus reducing the need for behavioural defence. The implications of individual differences in behavioural and structural defence are significant, as different relative investments in each may allow equal fitness outcomes for different behavioural or structural phenotypes. To our knowledge, no study has explicitly considered this question. Collectively, it highlights the need to better understand the role of structure in nest defence, while also focusing how individuals within a population vary in this defence.

Here we used redback spiders, *Latrodectus hasselti*, to study how web structure is affected by reproductive investment and the relative value of that investment, as well as whether individuals consistently vary in their investment in protective structures. Widow spiders (*Latrodectus* spp.) are ideal for this study as they build complex three-dimensional webs, which have distinct features that are used for foraging (gumfooted) as well as safety (structural lines) (Blackledge, Coddington, & Gillespie, 2003; Zevenbergen, Schneider, & Blackledge, 2008). Female spiders face predation pressure from conspecifics as well as from lizards and wasps (Blackledge et al., 2003; Trubl, Gburek, Miles, & Johnson, 2012), against which the dense three-dimensional webs are thought to defend against (Blackledge et al., 2003). And, while mature females have few parasites, eggcase parasites are common to widow spiders (Marie & Vetter, 2015; Pemberton & Rosa, 1940; Vetter et al., 2012) and spiders in general (Austin, 1985). Many widow spiders build a funnel-shaped retreat where they lay their eggcases (Shulov &Weissman, 1959), yet females appear to vary in the density of the retreat (N. DiRienzo, personal observation), or whether they even build a retreat (Barrantes & Eberhard, 2010). Thus, denser, more robust funnels may provide additional protection from parasitoids. Furthermore, although females respond to changes in body condition by altering their relative investment in different aspects of web structure for foraging (gumfooted lines) and safety (structural lines and web density), individual differences are still maintained across state differences (DiRienzo & Aonuma, 2017; DiRienzo & Montiglio, 2016b). These individual differences, coupled with limited plasticity, suggest that females may also show consistent differences in how they protect their reproductive investments.

In this study we asked the following three questions. (1) Do females increase investment in protective structures when they have an eggcase? (2) Does the level of protection provided structurally scale with the relative investment in the eggcase? (3) Are there consistent individual differences in web structure, including protective elements, when an eggcase is present versus absent. We predicted that females would increase funnel density, web mass (a proxy for web density), the number of structural lines, and become more aggressive when they have an eggcase versus when they do not. We also predicted that these same measures would positively scale with eggcase mass. Finally, we predicted that females would show consistent individual differences in all measures across eggcase status, thus suggesting they consistently vary in protective strategy.

## METHODS

We collected mature (*N* = 24) and immature (*N* = 7) female *L. hasselti* spiders in Fukuoka, Japan in the summer of 2015. The spiders were brought into the laboratory at Hokkaido University in Sapporo, Japan, after which they were assigned a unique identity number and placed in individual containers (7 cm high, 9 cm diameter). The spiders were provided two *Acheta domesticus* crickets per week, each approximately the same size as the spider. The spiders were maintained at 27 °C on a 12:12 h light:dark cycle. All mature spiders used in this experiment had mated in the field, as indicated by them laying viable eggcases throughout the experiment. The seven immature spiders were allowed to mature in the laboratory, after which they were successfully mated (i.e. all laid viable eggcases). These data are a subset of data collected as part of a larger experiment on *L. hasselti* (DiRienzo & Aonuma, 2017).

### Experimental Timeline

Each of the following behavioural and web assessment sequences lasted 2 weeks. Trials began by providing each spider a web-building structure and allowing them to build for 7 days (see Web Assessment methods below). After construction, we assayed aggression towards a prey cue, a method that positively correlates with prey capture success (DiRienzo, Montiglio, & Dornhaus, n.d.). The prey cue was presented three times per day for 3 consecutive days. We assessed web structure after the first round of behavioural trials in order to minimize handling effects on behaviour. After the third day, all spiders were removed from their webs and placed in their home containers for 5 days before beginning the next round of web building and behavioural trials. This process was repeated three times for field-mated spiders, and two times for laboratorymated spiders. Including the small number of mated spiders provides a control for any unknown age effects in the field-mated females, which were of an unknown age. We noted the presence or absence of an eggcase during each day of behavioural trials. All spiders were weighed both before being placed in the web-building structure and then again after all behavioural and web assays were complete but before being placed back in their home containers. All behavioural and web assays were conducted by N.D.

### Web Assessment and Eggcase Collection

We assessed individual web structure by providing each spider with a standardized structure to build upon. These structures consisted of a skeletonized cardboard box (24.5 × 19 × 10 cm). The box had three walls and all but 3 cm of the top removed, thus leaving a rectangular cardboard frame with the back, the bottom and the portion of the top walls remaining. This set-up provided a shelter along with a frame to build upon. The bottom and back walls were covered in black paper in order to ease the counting of individual web components. This box was placed inside a plastic container (40.7 × 28.5 × 185 cm), and the spider was given 7 days to construct a web. After the 7 days we removed the box and counted the number of gumfooted lines, as indicated by the sticky glue-like substance at the end, and structural lines, as indicated by those without the glue, connected to the floor. These methods have previously been used to assess black widow web structure (DiRienzo & Montiglio, 2016a, 2016b; Montiglio & DiRienzo, 2016). We also quantified the density of the funnel for each spider on a 0–3 scale. In this case, 0 corresponds to no funnel, 1 corresponds to a funnel outline consisting of only a few threads, 2 corresponds to a moderately dense funnel that still contains significant gaps (2+ mm), and 3 is a dense funnel with no significant gaps (<2 mm). Eggcases were removed before ranking in order to prevent observer bias. To check within-observer reliability, we first ranked each funnel in order of identity. We then immediately reranked all funnel densities in a randomized order relative to their identity. We used both measures to calculate the intraclass correlation coefficient (ICC), a measure of observer reliability, across the two rankings using the package ‘irr’ (Gamer, Lemon, Fellows, & Singh, 2012) in the software program R v.3.3.3 (R Core Team, 2015). We found that intraobserver agreement was highly consistent (ICC = 0.867, 95% CI = 0.842–0.889, *P* < 0.001), suggesting that the ranking measurements were highly reliable. All eggcases were weighed after removal, placed in a separate container with a unique identity corresponding to the female's identity, and tracked to ensure they were viable. All eggcases successfully hatched, indicating that the females were not egg dumping unviable eggs due to age or high food availability. Individual females laid between zero and three eggcases over the course of the experiment, with a mean of 1.9 eggcases per spider.

After all behavioural trials were complete, we removed the spider and subsequently gathered the web onto a plastic rod. The webs were then weighed using a Mettler Toledo XS3DU microbalance. In the North American congener, *Latrodectus hesperus*, web mass is highly correlated (*R*^2^ = 0.8) with web density (measured as the amount of reflectance from an illuminated web), and thus provides a measure as to the level of overall web investment (denser versus sparse webs) (Blackledge & Zevenbergen, 2007).

### Aggression Assay

After building a web for 7 days, we assessed spider aggression towards a vibratory cue simulating a prey item or intruder on the web. We removed any effects of live prey (DiRienzo, Pruitt, & Hedrick, 2013; Pruitt, Stachowicz, & Sih, 2012) by using a standardized vibrating mechanism (Classical Silicone Vibrator, Liler, Shenzhen, China). We attached a 10 cm long plastic cable tie to the end of the mechanism in order to reduce the intensity of the vibrations and the risk of damaging the web while also allowing us to apply the cue on specific silk lines. The vibrator provided 1 s pulses at 100 cycles/s separated by 0.5 s periods of reduced frequency. This pattern and frequency is the range of vibrations produced by prey items (Walcott, 1963), and has been shown to illicit a prey capture response in other widow spiders (DiRienzo & Montiglio, 2016a, 2016b; Montiglio & DiRienzo, 2016), and positively correlates with successfully capturing live prey (DiRienzo, Montiglio, & Dornhaus, n.d.). Furthermore, the behavioural response elicited by the vibratory cue is similar to that observed when any intruder is on the web, indicating that it may represent a more generalized form of aggression. We applied the cue three times per day: once within 1 cm of the retreat, once within 1 cm of the edge furthest from the retreat and once in between the two points. The cue was always presented for 15 s at each location, separated by 10 s intervals. We noted whether the spider attacked the prey cue and, if they did attack, whether they subsequently retreated to their funnel. The order of presentation was randomized for each spider. This process was repeated 24 h and 48 h later. All trials were conducted between 1000 and 1300 hours and in a randomized order within the day to minimize any potential influence of diel rhythms (Watts, Ross, & Jones, 2015).

### Statistical Analysis

We assessed the overall effect of eggcase presence on web structure and behaviour using linear mixed models and generalized linear mixed models. We modelled attack and retreat behaviour with logistic regression and logit links. Individual identity was included as a random effect, and spider mass, eggcase presence at the end of all trials, whether females were mated in the laboratory, web number and distance from the retreat where the stimulus was applied were all modelled as fixed effects. We modelled web structure in a similar manner, with the number of gumfooted lines, structure lines, web mass and funnel density (0–3) as response variables. Individual identity was again included as a random effect, and spider mass, eggcase presence, whether females were mated in the laboratory and web number were included as main effects. The number of gumfooted lines were modelled with Poisson error distributions, while web mass and funnel density were modelled with Gaussian distributions. We accounted for overdispersion in Poisson models by including an observation-level random effect (OLRE) (Harrison, 2014). All models were fitted with the ‘lme4’ package (Harrison, 2014) in R. We centred spider mass and distance to a mean of zero and standard deviation of one before fitting all models.

We assessed the relationship between the reproductive investment and protective investment via changes in behaviour and web structure using generalized linear mixed models. All response variables and random effects were the same as before, but with only a main effect of the percentage mass of the eggcase relative to the female's body mass and whether females were mated in the laboratory. Thus, eggcase percentage represents the relative investment in reproduction. The models were only fitted to the subset of data consisting of females that laid eggcases during that instance of web building and behavioural trials. Overfitting of models was not a concern as there were a total of 52 eggcase observations and 80 total observations, while our largest model contained one random effect and four main effects. This falls under the 10 observations per parameter that is generally suggested (Harrell, 2015).

Finally, we assessed the repeatability of all behavioural and web measures, as well as of reproductive output, using the R package ‘rptR’ (Schielzeth & Nakagawa, 2011). All repeatability calculations were adjusted based on the main effects previously described. Adjusted repeatabilites allow for the calculation of repeatability after controlling for potentially confounding covariates such as differences in body size, sex or nutritional status (Nakagawa & Schielzeth, 2010). We allowed rptR to calculate 95% confidence intervals via parametric bootstrapping procedures (*N* = 1000). Repeatability values whose confidence intervals did not overlap zero were deemed to be statistically significant.

## RESULTS

**Figure 1:**
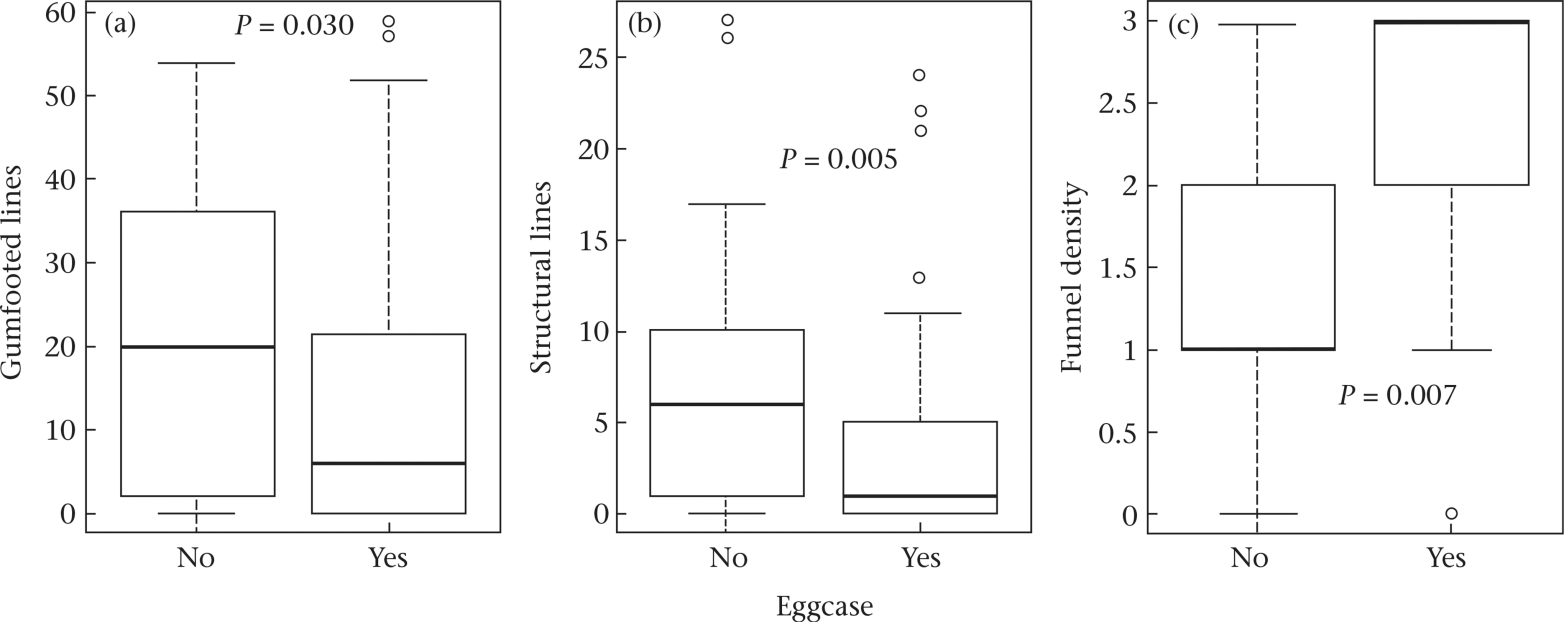
Effect of eggcase presence on (a) the number of gumfooted lines built, (b) the number of structural lines built and (c) funnel density. Box plots show 25% and 75% quartiles (boxes), medians (lines in the boxes), outermost values within the range of 1.5 times the respective quartiles (whiskers) and outliers (circles).

Overall, females that had an eggcase were not significantly more aggressive or likely to retreat than those who did not (attack: β = 0.313 ± 0.447, *P* = 0.483; retreat: β = −0.128 ± 0.528, *P* = 0.808; Table A1). Yet, females did alter web structure in response to having an eggcase. Females built fewer structural (β = −1.124 ± 0.400, *P* = 0.005) and gumfooted lines (β = −1.362 ± 0.626, *P* = 0.030) but denser protective funnels (β = 0.681 ± 0.253, *P* = 0.007) when they had an eggcase (Fig. 1, Tables 1, 2). We found no differences in the overall web mass (β = −0.379 ± 0.298, *P* = 0.203; Tables 1, 2, Table A1).

Females showed moderate to high repeatabilities in all behavioural measures (range 0.164–0.492) and web measures (range 0.388–0.789) (Table 3) (Bell, Hankison, & Laskowski, 2009). Thus, females showed consistent individual differences in these traits across the successive trials.

**Figure 2:**
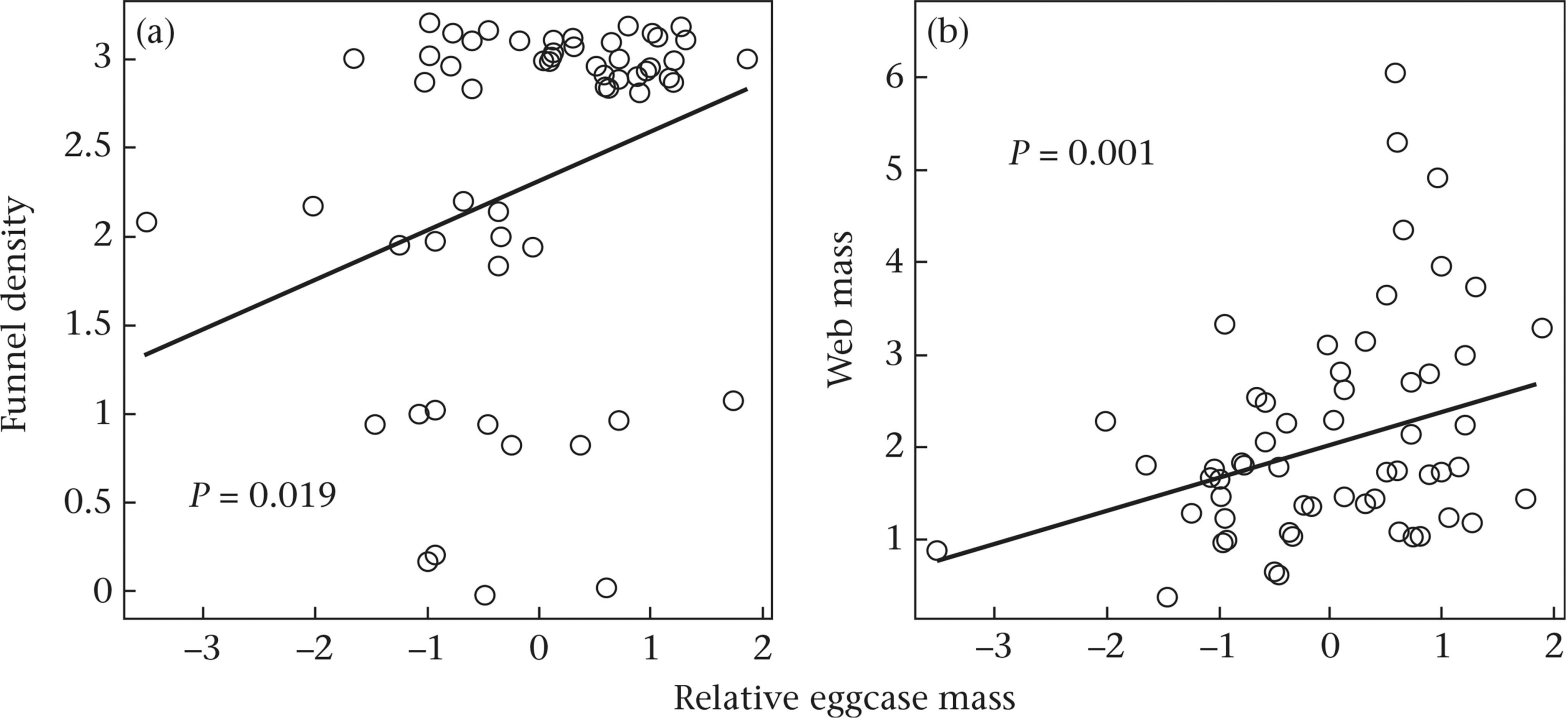
Relationship between relative eggcase mass and (a) investment in the funnel and (b) overall web mass.

We found evidence that females differed in their web investment in response to the relative eggcase mass. Both funnel density and the overall web mass were positively related to relative eggcase mass (the percentage of body mass contributed to the eggcase) (funnel density: β = 0.276 ± 0.105, *P* = 0.008; web mass: β = 0.358 ± 0.153, *P* = 0.020; Fig. 2, Table 4). Yet, neither gumfooted lines, structural lines, nor either behavioural measure was related to relative eggcase mass (gumfooted lines: β = 0.236 ± 0.326, *P* = 0.468; structural lines: β = −0.292 ± 0.218, *P* = 0.180; attack: β = 0.027 ± 0.238, *P* = 0.910; retreat: β = −0.352 ± 0.221, *P* = 0.111; Tables A2, A3).

Finally, spider mass was positively related to absolute eggcase mass, indicating that larger spiders laid larger eggcases (spider mass: β = 29.434 ± 1.814, *P* < 0.001; Table A4). Furthermore, absolute eggcase mass was repeatable (0.546, 95% CI = 0.222–0.765; Table 3), even after controlling for body size differences. Thus, females showed individual differences in their relative reproductive output.

Females being mated in the field or in the laboratory, a rough proxy for female age, was significant only when predicting the relationship between web mass and relative eggcase investment. Specifically, females who were mated in the laboratory built heavier webs (β = 1.114 ± 0.472, *P* = 0.018; Table 4).

## DISCUSSION

Our goal here was to determine how females alter aspects of behaviour and web structure in response to having an eggcase, and whether those changes correlate to relative investment in the eggcase. Our results indicate that, as predicted, females alter web structure when they are in possession of an eggcase by building fewer structural and gumfooted lines, but a denser protective funnel. Yet, no differences were seen in their aggressive behaviour. Furthermore, our prediction that females should scale protective investment with reproductive investment was supported, as both funnel density and web mass were positively correlated with relative eggcase mass. Interestingly, despite these positive relationships, fixed effects, including eggcase, predicted relatively little variation, while the individual random effect predicted extensive variation. This individual variation drove moderate to very high repeatability across all measures of behaviour, web structure and reproductive output.

Much of the work investigating how organisms protect reproductive investments has focused on behavioural mechanisms (Andersson et al., 1980; Burtka & Grindstaff, 2013; Greig-Smith, 1980; Hollander et al., 2008), yet here we demonstrate that aspects of the extended phenotype may be equally, if not more, important in some systems. The strong effects of eggcase presence on funnel density suggest that funnel density is primarily related to offspring protection rather than personal protection. Although *Latrodectus* eggcases have few natural predators, they do have a range of parasites (Bianchi, 1945; Eason, Peck, & Whitcomb, 1967; Pemberton & Rosa, 1940; Vetter et al., 2012), and it has been observed that females may steal eggcases and add them to their own web, potentially to dilute parasite pressure (Downes, 1984). Forming an extremely dense cloud of silk directly around the eggcase may minimize the angle of approach for such parasites and conspecifics, likely increasing the female's ability to defend the eggcase. Interestingly, we saw a negative effect of eggcase presence on the number of structural lines connecting to the ground, which goes counter to the argument that such lines aid in protection (Blackledge et al., 2003; DiRienzo & Montiglio, 2016a, 2016b). Such an argument may indeed be true if the structural lines provide protection to the female but not to the offspring. This line of reasoning is reinforced by the fact that production of both eggcases and webs are energetically costly (Ford, 1977; Jakob, 1991), and thus females may make trade-offs by investing more in structures that provide a greater reproductive fitness benefit (funnel) and investing less in others (structural lines). Overall the plastic response of structural elements with clear protective benefits highlights the critical role of extended phenotypic traits in mediating reproductive success.

Counter to predictions, we did not see a change in female aggression, and we saw a reduction in gumfooted lines after females laid an eggcase. This outcome has two possible explanations. One is that the aggression assay used does not translate to active anti-predator/parasite defence outside of the funnel, and thus eggcase presence did not alter the response. The alternative, and more likely explanation, is that the lack of response actually represents an increase in eggcase guarding within the funnel. Studies in *Latrodectus* spiders have shown that weight loss leads to increases in aggression towards vibratory cues and building of gumfooted lines (DiRienzo & Montiglio, 2016b; DiRienzo, Montiglio, & Dornhaus, n.d.; Zevenbergen et al., 2008). Given that females who laid an eggcase had a mean mass loss of 50% (range 36–62%), while those who did not had a mean loss of 13% (range 4–27%), we would expect aggression to significantly increase in those who laid an eggcase. The lack of an increase in aggression or gumfooted lines suggests that females dedicated more time to eggcase guarding in the funnel and less time to foraging or actively defending their web. Indeed, we frequently observed eggcase guarding, as indicated by the female perching on the eggcase and attempting to bite the forceps when we attempted to remove an eggcase, and such eggcase guarding is a common defence mechanism in spiders (Barrantes & Weng, 2007; Fink, 1987; Horel & Gundermann, 1992). Future studies will investigate eggcase guarding more explicitly as well as how such defensive behaviours interact with extended phenotypic traits to determine the relative contribution of each to individual fitness outcomes.

Theory predicts that organisms should scale their energy expenditure in accordance with the value of their offspring, driving parents to more vigorously defend larger or older offspring (Andersson et al., 1980; Greig-Smith, 1980; Montgomerie & Weatherhead, 1988). Our results provide evidence that this scaling might not occur at the behavioural level, but instead at a structural level. Both web mass and funnel density were positively related to relative eggcase mass. Unexpectedly, the number of structural lines connecting to the ground did not scale with eggcase mass. The increase in web mass without an increase in lines connecting to the ground suggest that females are increasing overall web density, as web mass is strongly correlated with web density (*R*^2^ = 0.8) (Blackledge & Zevenbergen, 2007). Thus, females appear to expend additional energy to increase web and funnel density when they have a large relative reproductive investment. While we are unaware of the history of those females that mated in the field, and thus do not know their age, mating history or number of eggcases laid prior, the general lack of a difference between them and those mated in the laboratory suggests that those factors are not highly relevant to how web structure and behaviour vary in response to reproductive investment. Still, a more tightly controlled experiment is needed to disentangle any possible effect of these factors. For example, if young females are able to more vigorously defend behaviourally, they may have less need to protect via web structure. Future projects will investigate how additional state variables such as age, condition and experience influence relative reproductive investment and the associated shifts in web structure.

Despite the relationships we found between eggcase properties and web structure, both eggcase presence and relative eggcase weight explained relatively little of the variation in web structure and behaviour. Specifically, the fixed effects, including eggcase presence or relative mass, explained 5–23% of the variation in web structure and behaviour, while the associated random effect explained 46–65% of the variation (Tables 1, 2, 4). Thus, although individuals plastically respond to their state via web structure, which is a known phenomenon (Blamires, Hasemore, Martens, & Kasumovic, 2017; DiRienzo & Aonuma, 2017; DiRienzo & Montiglio, 2016b), in the present study, much more of the variation in these traits was due to individual variation. This result is reinforced by the fact that there were high to extremely high repeatability values for the structural components, ranging from 0.388 to 0.789, and moderate to high repeatability in behavioural components, ranging from 0.164 to 0.492. This implies that, although plasticity is present, individuals' behavioural and web-building tendencies are fixed and there are limits to how much plasticity one can express. The relative inflexibility that underlies individual differences may drive trade-offs among individuals whereby some individuals may excel at foraging, while others are better able to protect their reproductive investment. Furthermore, these individual differences were also mirrored in the mass of the eggcase. Thus, these trade-offs may span multiple aspects of the organism's phenotype, from behaviour to web structure and reproductive output. Different combinations of these traits all may potentially produce equal fitness outcomes (Pruitt, Bolnick, Sih, DiRienzo, & Pinter-Wollman, 2016; Pruitt et al., 2017), which in turn may maintain trait variation over time. Collectively, this highlights the need to take a multitrait approach to understanding how individual variation in multiple distinct traits interact to influence organism fitness.

## Supplementary Material

supplement

## Figures and Tables

**Table 1:** Generalized linear mixed model outputs for the number of gumfooted lines and structural lines

	Gumfooted lines				Structural lines			
**Random effects**	**Estimate**				**Estimate**			
Identity	1.493				2.028			
OLRE	2.290				0.345			
**Fixed effects**	**β**	**SE**	***Z***	***P***	**β**	**SE**	***Z***	***P***
Intercept	3.945	0.747	5.298	<0.001	1.850	0.524	3.529	<0.001
Web number	−0.626	0.251	−2.495	0.013	−0.179	0.134	−1.336	0.182
Spider mass	0.224	0.274	0.818	0.414	0.120	0.173	0.687	0.492
Eggcase	−1.362	0.626	2.175	0.030	−1.124	0.400	−2.813	0.005
Mated in lab	−1.084	0.751	−1.445	0.149	−0.691	0.694	−0.995	0.317
Marginal *R*^2^	0.163				0.079			
Conditional *R*^2^	0.475				0.724			

OLRE: observation-level random effect. Spider mass was centred to a mean of zero and standard deviation of one before fitting. Eggcase possession was measured by a 0/1 response. A total of 79 webs were measured from 31 individuals.

**Table 2:** General linear mixed model outputs for the funnel density and web mass

	Funnel density				Web mass			
**Random effects**	**Estimate**				**Estimate**			
Identity	0.527				0.833			
Residual	0.382				0.513			
**Fixed effects**	**β**	**SE**	***t***	***P***	**β**	**SE**	***t***	***P***
Intercept	1.617	0.310	5.209	<0.001	2.376	0.370	6.426	<0.001
Web number	−0.021	0.092	−0.233	0.815	−0.040	0.106	−0.376	0.707
Spider mass	0.256	0.112	2.278	0.023	0.556	0.132	4.289	<0.001
Eggcase	0.681	0.253	2.692	0.007	−0.379	0.298	−1.273	0.203
Mated in lab	0.098	0.352	0.280	0.780	0.794	0.433	1.833	0.067
Marginal *R*^2^	0.204				0.229			
Conditional *R*^2^	0.665				0.706			

Spider mass was centred to a mean of zero and standard deviation of one before fitting. Eggcase possession was measured by a 0/1 response. A total of 79 webs were measured from 31 individuals.

**Table 3:** Adjusted repeatability values and associated 95% confidence for all web, behavioural and reproductive traits

Trait	Repeatability	95% CI
Gumfooted lines	0.388	0.022–0.662
Structural lines	0.789	0.494–0.933
Funnel density	0.580	0.356–0.768
Web mass	0.619	0.426–0.785
Attack	0.492	0.270–0.681
Retreat	0.164	0.024–0.330
Eggcase mass	0.546	0.222–0.765

**Table 4:** General linear mixed model outputs for the funnel density and web mass of web made by spiders who laid an eggcase

	Funnel density				Web mass			
**Random effects**	**Estimate**				**Estimate**			
Identity	0.547				0.657			
Residual	0.290				0.763			
**Fixed effects**	**β**	**SE**	***t***	***P***	**β**	**SE**	***t***	***P***
Intercept	2.305	0.181	12.717	<0.001	2.007	0.221	9.092	<0.001
Relative eggcase mass	0.276	0.105	2.633	0.008	0.358	0.153	2.332	0.020
Mated in lab	0.322	0.378	0.853	0.393	1.114	0.472	2.361	0.018
Marginal *R*^2^	0.107				0.199			
Conditional *R*^2^	0.690				0.569			

The relative eggcase mass was centred to a mean of zero and standard deviation of one before fitting. A total of 59 webs were measured from 28 individuals.

## Appendix

**Table A1:** Generalized linear mixed model outputs predicting attack and subsequent retreating behaviour

	Attack				Retreat			
**Random effects**	**Estimate**				**Estimate**			
Identity	5.138				1.522			
**Fixed effects**	**β**	**SE**	***Z***	***P***	**β**	**SE**	***Z***	***P***
Intercept	3.063	0.771	3.972	<0.001	−23.172	0.796	3.987	<0.001
Web number	−0.119	0.159	−0.747	0.455	−0.124	0.198	−0.627	0.531
Spider mass	−0.018	0.217	0.081	0.935	−0.206	0.221	−0.930	0.353
Eggcase	0.313	0.447	0.701	0.483	−0.128	0.528	−0.243	0.808
Distance	−0.702	0.157	−4.463	<0.001	0.766	0.195	3.925	<0.001
Mated in lab	−0.459	1.015	−0.452	0.651	−0.016	0.724	−0.022	0.982
Marginal *R*^2^	0.046				0.087			
Conditional *R*^2^	0.628				0.376			

Spider weight was centred to a mean of zero and standard deviation of one before fitting. Eggcase presence was measured as a 0/1 response. A total of 611 observations of attack behaviour were made from 31 individuals, while 459 observations of retreating behaviour were measured from 30 individuals.

**Table A2:** Generalized linear mixed model outputs for the number of gumfooted lines and structural lines made by spiders who laid an eggcase

	Gumfooted lines				Structural lines			
**Random effects**	**Estimate**				**Estimate**			
Identity	3.470				3.129			
OLRE	1.151				0.313			
**Fixed effects**	**β**	**SE**	***Z***	***P***	**β**	**SE**	***Z***	***P***
Intercept	1.304	0.406	3.215	0.001	0.018	0.473	0.038	0.970
Relative eggcase mass	0.236	0.326	0.762	0.468	−0.292	0.218	−1.340	0.180
Mated in lab	−1.012	0.900	−1.131	0.258	0.287	0.901	0.319	0.750
Marginal *R*^2^	0.039				0.022			
Conditional *R*^2^	0.264				0.768			

OLRE: observation-level random effect. Relative eggcase mass was centred to a mean of zero and standard deviation of one before fitting. A total of 59 webs were measured from 28 individuals.

**Table A3:** Generalized linear mixed model outputs predicting attack and subsequent retreating behaviour

	Attack				Retreat			
**Random effects**	**Estimate**				**Estimate**			
Identity	3.476				0.760			
**Fixed effects**	**β**	**SE**	***Z***	***P***	**β**	**SE**	***Z***	***P***
Intercept	1.503	0.451	3.337	<0.001	−2.035	0.496	−4.107	<0.001
Relative eggcase mass	0.027	0.238	0.113	0.910	−0.352	0.221	−1.596	0.111
Mated in lab	0.368	0.989	0.392	0.695	−0.575	0.744	−0.773	0.440
Marginal *R*^2^	0.000				0.042			
Conditional *R*^2^	0.514				0.213			

Relative eggcase mass was centred to a mean of zero and standard deviation of one before fitting. Eggcase presence was measured as a 0/1 response. A total of 432 observations of attack behaviour were made from 28 individuals, while 345 observations of retreating behaviour were made from 27 individuals.

**Table A4:** Generalized linear model predicting the relationship between female body size and eggcase weight

	Eggcase weight			
**Random effects**	**Estimate**			
Identity	110.370			
Residual	91.660			
**Fixed effects**	**β**	**SE**	***t***	***P***
Intercept	73.005	2.753	26.519	<0.001
Spider mass	29.434	1.841	15.989	<0.001
Mated in lab	1.877	5.751	0.326	0.774
Marginal *R*^2^	0.787			
Conditional *R*^2^	0.901			

A total of 58 eggcases were laid by 28 individuals over the course of the experiment.
